# Measurement and Comparison of Melt-Blowing Airflow Fields: Nozzle Modifications to Reduce Turbulence and Fibre Whipping

**DOI:** 10.3390/polym13050719

**Published:** 2021-02-26

**Authors:** Ying Yang, Yongchun Zeng

**Affiliations:** College of Textiles, Donghua University, Shanghai 201620, China; yangying@mail.dhu.edu.cn

**Keywords:** nozzle modification, turbulence control, hot-wire measurement, melt-blowing, fibre oscillation

## Abstract

In the melt-blowing process, micro/nanofibrous nonwovens are attenuated and formed through aerodynamic force in a turbulent airflow field. In this work, two types of airflow-directors were added under a common melt-blowing slot-die nozzle to obtain modified airflow fields. The effect of airflow-directors on time-averaged characteristics, turbulence intensity, and temperature fluctuation intensity are achieved through the simultaneous measurement of fluctuating velocity and fluctuating temperature using a two-wire probe hot-wire anemometer. Moreover, the influence of airflow-directors on fibre oscillations are also investigated through high-speed photography. The distribution of turbulence intensity and temperature fluctuation intensity reveals the characteristics of fluctuating airflow fields formed by different melt-blowing slot-die nozzles. Through the analyses of airflow characteristics and fibre oscillations, we can find that the arrangement of airflow-directors has a great impact on both turbulence distribution and fibre oscillation.

## 1. Introduction

Due to it becoming a popular method due to its application on producing high-efficiency air filters in industry [1,2,3,4], melt-blowing technology has been of increasing interest in recent years owing to the development of nanotechnology [5,6,7,8,9]. During the melt-blowing process, polymer melts are attenuated into micro/nanofibrous nonwovens with the help of high-speed hot air jets [10]. Commercially, the melt-blowing slot-die, which contains a nozzle with a pair of slots to eject the two inclined hot air jets, is most commonly used to rapidly stretch the molten strand of polymer. As an aerodynamically-driven process, the key defect during production (nonuniform fibre diameters and fibre whipping) are assumed to be rooted in the aerodynamics of the turbulent airflow field [11,12,13]. The complex turbulent airflow field of melt-blowing, however, makes it difficult to fully reveal the underlying mechanism. In addition, the melt-blowing airflow field has been studied by researchers for decades and the effects of air turbulence on the fibre motion remain elusive. Therefore, to better control the fluctuating airflow field and to achieve a more stabilized pattern of fibre oscillation, the studies of the aerodynamics and fibre motion pattern in the melt-blowing airflow field are of great importance.

There are two ways to investigate the melt-blowing airflow fields: Experimental and computational fluid dynamics (CFD). The experimental work in the literature mainly focuses on the measurement of mean velocity field and mean temperature field [14,15,16,17,18]. In these researches, the mean velocity was obtained through a Pitot tube, while the mean temperature was measured through a thermocouple. Based on the experimental data, they developed correlations to predict the airflow fields below these nozzles. However, as the melt-blowing air is turbulent, we believe measurements which obtain only the mean airflow characters are still inadequate and not representative. CFD is the other widely used way to investigate the melt-blowing airflow field [8,19,20,21], owing to its advantage of saving costs. Tan et al. performed the simulation work of the airflow field formed by the slot-die nozzle with a laval add-on device [22]. Hassan et al. modified the slot-die nozzle with air constrictors to help maintain the centreline velocity and temperature [23]. There are two main targets in these researches: Maintaining the centreline velocity and stabilizing the turbulence. On the one hand, by maintaining high centreline air velocity and keeping the temperature near the nozzle exit can help to achieve higher fibre attenuation [23]. On the other hand, turbulence intensity along the centreline must be minimized, since strong velocity fluctuations in the airflow field can cause fibre breakup or stick to the die face [24]. Thus, turbulent characteristics are calculated through some of the CFD simulation works [12,25,26]. However, due to inconvenience and complicity, investigations of turbulent characteristics in the melt-blowing airflow field based on turbulence measurements have rarely been performed. Our previous work [27] introduced the hot-wire anemometer (HWA) with a single hot-wire to measure the turbulent airflow field under the melt-blowing slot-die nozzle. In another recent work [28], we improve the separately measured experiment into simultaneously measured results of real-time velocity and temperature through a two-wire HWA.

As to researches of fibre oscillation (often referred to as ‘whipping’ in the literature), it was first observed by Shambaugh and coworkers though multiple-expose [29,30]. Both the experimental results appear to be a bundle of jets rather than a single jet. In spite of the high-speed camera applied to record the melt-blowing process, it was not until Beard and Shambaugh’s recording that the trajectory for single fibre was captured [31]. Xie and Zeng also applied the high-speed camera to record successive images of fibre path [32]. There experiment show that the fibre whipping motion sets in as soon as the polymer jet begins issuing from the nozzle exit. According to the observations, Yarin and coworkers described the phenomenon as ’bending instabilities’ and developed a quasi-one-dimensional model according to the effect of lift force and drag fore to describe the fibre lay-down pattern in melt-blowing [33]. Kumar and coworkers further developed this model and applied a time-varying cosine function as turbulent fluctuation at the nozzle inlet [34]. Their results suggest that oscillations would arise from turbulent fluctuations in the surrounding air. However, the airflow field applied to the quasi-one-dimensional model was a basic model of a single round jet. Thus, it can hardly apply to the effect of nozzle configuration on the fibre motion without establishing the complex airflow theories for different nozzles.

The present work discusses the effect of the airflow-directors on the pattern of the fluctuating airflow field and on the fibre oscillation pattern under the melt-blowing slot-die nozzles. The airflow-directors are intended to add boundaries at the inner/outer space between the melt-blowing air jets. The non-isothermal turbulent airflow fields are measured through an X-wire HWA, which can provide real-time velocity and temperature simultaneously. According to the time-averaged results, the velocity and temperature distributions of different nozzle configurations are compared. The turbulent characteristics in the x−z plane, including the turbulence intensity and temperature fluctuation intensity are obtained and analysed. Further, to compare the effect of nozzle configuration on the fibre motion under the melt-blowing slot-die, the characteristics of fibre oscillation under the nozzles are analyzed based on high-speed photography.

## 2. Experimental Setup

### 2.1. Schematic of Melt-Blowing Device

**Figure 1:**
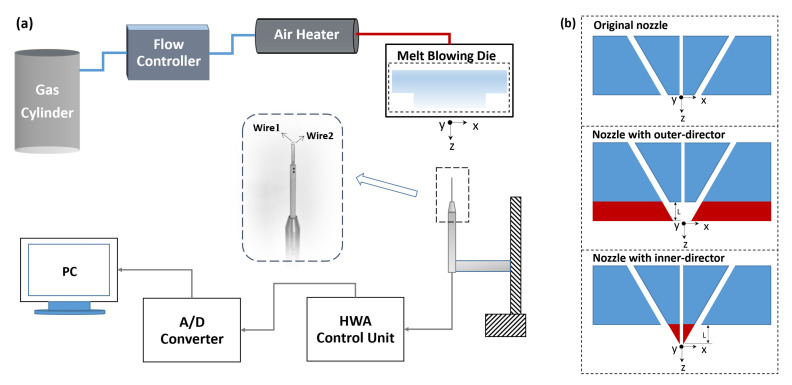
(**a**) Schematic of melt-blowing device and hot-wire measuring system. (**b**) Schematic nozzle configurations (cross-sectional view).

The schematic of the melt-blowing device and the hot-wire system is shown in Figure 1a. The air issuing from the melt blowing slot-die nozzle is provided by a gas cylinder with the flow rate controlled by a flow controller while the flow temperature is controlled by an air heater. The hot-wire measuring system includes a hot-wire anemometer (HWA) control unit (Dantec StreamLine CTA90C10 and Dantec StreamLine 90C20, Dantec Dynamics, Skovlumde, Denmark), an A/D Converter (WS-5921, Beijing Wave Spectrum, Beijing, China), and a traversing unit. The airflow field, which includes air velocity and temperature of each measuring positions, are obtained simultaneously.

During the experiment, 140 °C-air was supplied to the dual-slots with a flow rate of 3 g/min, which created an air velocity of 150 m/s at the nozzle exit. The die temperature was set to be the same as the air to avoid heat transfer between the air jets and the nozzle. The schematics of the three nozzles compared in this work, the original slot-die nozzle [28], the nozzle with outer airflow-director, and the nozzle with inner airflow-director, are presented in Figure 1b. The original nozzle, which is part of a common slot-die used in our previous experiment, possesses two 0.56 mm-slots with a slot angle of 30° and a slot length of 1.28 mm. Based on the original nozzle, outer airflow-director and inner airflow-director are added to lead the high-speed air to further downstream. For both the outer and inner directors, the add-on part of the nozzle (colored in red in Figure 1b) is with a thickness of 0.74 mm. To be mentioned, the inner/outer directors represent the solid boundaries added at the inner/outer side to the originally unbounded jets. The influence of manufacturing precision was not considered as the nozzles were manufactured under the same precision (surface roughness Ra 0.8, cutting precision IT7).

The coordinate system used for different nozzles are also shown in Figure 1a,b, where the origin of the coordinate system is located on the axis of symmetry right below the nozzle. The *z* direction is directed vertically downward along the spinning line, while the *x* direction is parallel to the face of the nozzles. The *y* direction is perpendicular to the plane of Figure 1b.

### 2.2. Instantaneous Velocity and Temperature Measurements

**Figure 2:**
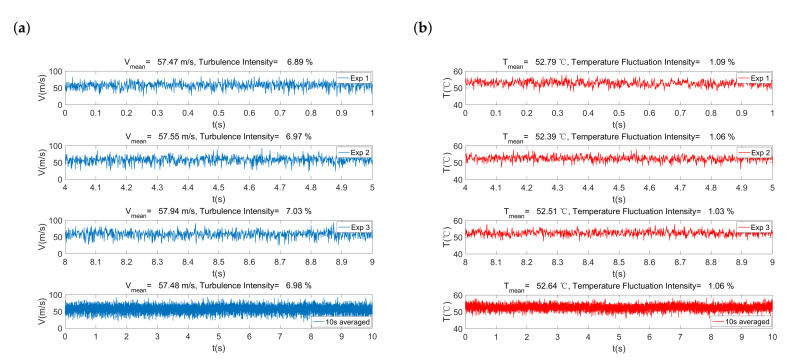
Example of instantaneous signal measurements at z=24.5 mm under the original slot-die nozzle: (**a**) Instantaneous velocity and (**b**) instantaneous temperature.

Through the HWA measurements, the real-time signals of both velocity and temperature are obtained at a sampling frequency of 1024 Hz simultaneously with a two-wire probe. Thus, the experiment data are repeatedly measured 10,240 times during the 10 s interval at each targeted measuring position. To illustrate the measurements in this work, the instantaneous velocity and temperature signals at the position z=24.5 mm under the original slot-die nozzle are presented in Figure 2a,b. It can be seen in Figure 2a that the instantaneous velocity fluctuates temporally in the turbulent airflow field. The mean velocities $V_{mean}$ calculated by of the instantaneous signals during 1st s, 5th s, and 9th s are 57.47 m/s, 57.55 m/s, and 57.94 m/s, respectively. However, the mean velocity in this work is obtained in a time-averaged way base on the 10 s interval to minimize the measurement uncertainties. Similarly, the result of turbulence intensity, mean temperature, and temperature fluctuation intensity are also obtained in a time-averaged way in this work.

### 2.3. Calibration

**Figure 3:**
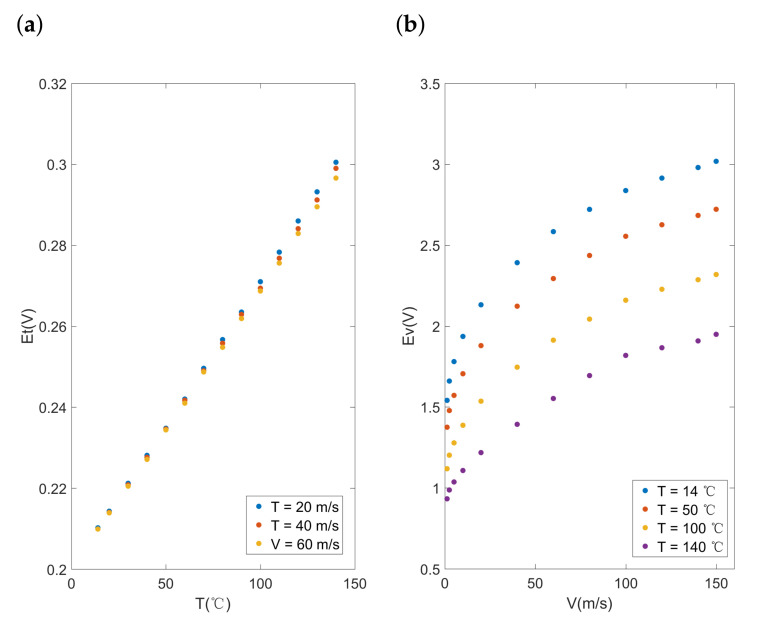
Calibration curves: (**a**) Temperature sensor response and (**b**) velocity sensor response.

The calibration process of the X-wire miniature HWA probe (55p61, Dantec Dynamics, Skovlumde, Denmark) was made before the measurement. The two-wire probe was placed below a round nozzle with an exit diameter of 5 mm, where the turbulence level was minimized. The air applied to the nozzle exit was set over the velocity range of interest (1.25–150 m/s) under different temperature settings (across the range 14–140 °C). As the two-wire probe include two sensors that output both velocity and temperature signals, velocity and temperature calibration were performed under each given velocity and temperature. For the temperature sensor, the sensor response to temperature T can be described as a linear function:

(1) $$E_{t}=k_{0}+k_{1}\cdot T,$$

where $k_{0}$ and $k_{1}$ are temperature wire calibration coefficients. This linear correlation can be further verified according to the calibration data show in Figure 3a. While for the velocity sensor, the sensor response to velocity can be fitted into a polynomial curve in 4th order:

(2) $$U=C_{0}+C_{1}\cdot E_{comp}+C_{2}\cdot E_{comp}^{2}+C_{3}\cdot E_{comp}^{3}+C_{4}\cdot E_{comp}^{4},$$

where $C_{0}$ , $C_{1}$ , $C_{2}$ , $C_{3}$ , and $C_{4}$ are velocity wire calibration coefficients. $E_{c}omp$ is the compensated voltage of velocity sensor, which must be converted with ambient temperature through the following equation:

(3) $$E_{comp}=\sqrt{\frac{T_{w}-T_{0}}{T_{w}-T_{a}}}\cdot E_{v},$$

where $E_{v}$ is the measured velocity signal voltage, $T_{w}$ is the constant wire temperature, i.e., 217 °C, $T_{a}$ is the air temperature of the target position, and $T_{0}$ is the temperature of the calibration curve. It should be noted that the velocity wire calibration can be strongly influenced by temperature and it is suggested in the literature that the equation of compensation can be used as $T_{a}-T_{0}\leq \pm 5$ °C. Thus, to improve the compensation of velocity signal, the calibration curves were repeatedly obtained at every 10 °C increasement of temperature. As a result, 14 curves rather than 1 curve were applied to ensure calibration accuracy in the non-isothermal melt-blowing airflow field. Figure 3b present the velocity sensor response to velocity under a different calibration temperature. This further verifies the importance of temperature compensation to velocity sensor.

## 3. Measurement Results

### 3.1. Time-Averaged Velocity and Temperature

**Figure 4:**
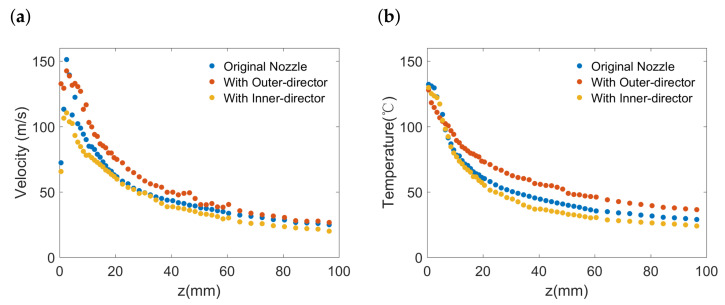
Centreline decay of (**a**) velocity and (**b**) temperature.

Figure 4 shows how the airflow-director affects the centreline velocity and temperature along the *z*-axis. It can be seen in Figure 4a that, although supplied with the same flow rate of air, the centreline velocity peak for the nozzle with the inner-director is lower. This phenomenon indicates that, even with the same consumption of energy, the air jet momentum distribution can be affected by the existence of an inner airflow-director. Figure 4a also shows that the presence of an outer airflow-director can help maintain the centreline velocity at a higher value at some distance downstream compared with the original nozzle.

Furthermore, Figure 4b shows the impact of airflow-directors on the temperature along the *z*-axis. The temperature decay for the nozzle with outer airflow-director is considerably slower than that of the original nozzle, indicating that the existence of outer airflow-director can retard the temperature decrease along the centreline. However, the centreline temperature for the nozzle with inner airflow-director resembles that of the original nozzle.

Based on the results, we can find that the presence of the outer airflow-director not only maintains the air velocity at the centreline but also help to slow down the decrease of temperature. This probably due to the presence of the outer airflow-director can act as barriers besides the jet, which can prevent ambient air entrainment near the nozzle exit, where both air velocity and air temperature are of the highest value.

### 3.2. Turbulence Intensity

**Figure 5:**
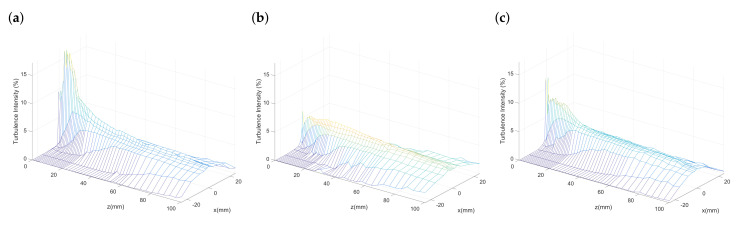
Mesh plots of turbulence intensity: (**a**) Original nozzle, (**b**) nozzle with outer-director, and (**c**) nozzle with inner-director.

Apart from the time-averaged flow behaviors, the turbulent behaviors for different nozzles are also obtained in our work. Figure 5 presents the turbulence intensity in the *x*-*z* plane where the coordinates are bounded by x=25 mm and z=100 mm. The turbulence intensity is calculated by the ratio of root-mean-square of turbulent velocity fluctuations $U_{rms}$ and the nozzle exit velocity $U_{0}$, where $U_{rms}=\sqrt{\frac{1}{N-1}\sum_{i=1}^{N}{\left(U_{i}-U_{mean}\right)}^{2}}$. A finer mesh of measuring points is specified in the region near the nozzle exit to provide a more detailed fluctuating velocity and temperature. Thus, the positions of the nodes in the mesh plot are arranged in a nonuniform way. It can be seen that, for all nozzle configurations, the turbulence intensity increase along the centreline and then decrease after it reaches a maximum. Apart from the centreline, the sharp turbulence decrease in the lateral direction (*x*) is also shown in Figure 5a–c. The peak of turbulence intensity for the nozzle with the outer-director is of the lowest value among the three conditions, indicating the dramatically intensified fluctuating velocity near the nozzle exit can be decreased through the add-on of an outer airflow-director.

**Figure 6:**
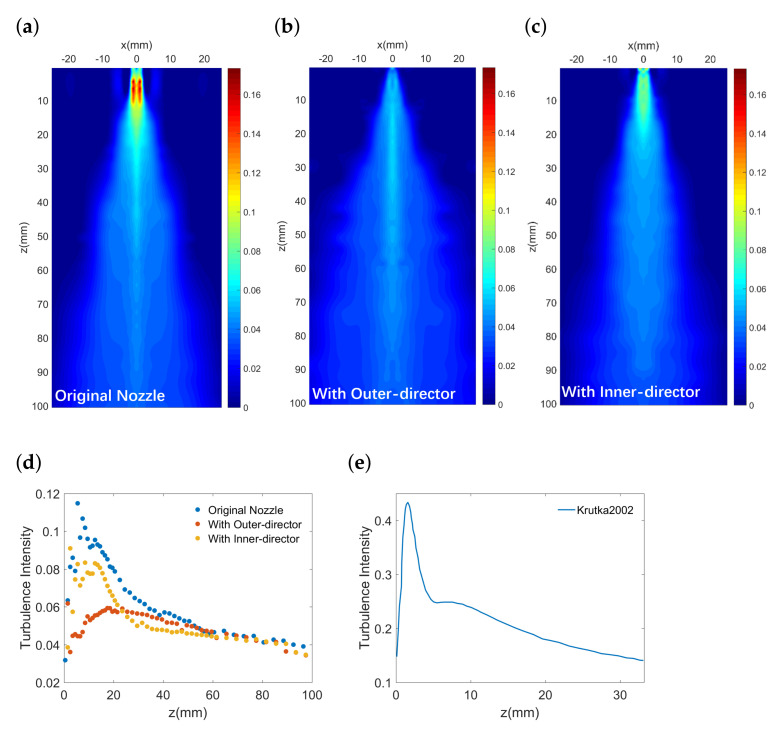
(**a**) Distribution plot of turbulence intensity under original nozzle. (**b**) Distribution plot of turbulence intensity under nozzle with outer-director. (**c**) Distribution plot of turbulence intensity under nozzle with inner-director. (**d**) Centreline turbulence intensity, and (**e**) Calculated centreline turbulence intensity in literature123 [12] (for comparison).

To further explore the effect of airflow-directors on turbulence intensity distribution by comparing with the original nozzle [28], we obtain the distribution plot of turbulence intensity through biharmonic spline interpolation in Figure 6. This is based on the densely distributed experimental data shown in Figure 5. The positions with high turbulence intensities can be identified by the light blue region together with the region marked as yellow and red (around over 6%). Accordingly, the region colored dark blue represents a low turbulence intensity level (below 1%). The region colored in shallow blue corresponds to medium turbulence intensity level (between 1% and 6%).

It can be seen in Figure 6a that the turbulence distribution for the original nozzle exhibit a pattern that the region with a high turbulence level (colored in red, yellow, and light blue) shows up around the centreline in the near field (0 mm <z< 20 mm). We observe no such pattern in the same region for the nozzle with the outer-director (Figure 6b), while a similar pattern colored in light blue can be found in Figure 6c. This manifests that the high turbulence level below the nozzle exit can be decreased by adding an outer airflow-director. Furthermore, in the far field (z> 20 mm), there is a larger area colored in shallow blue for the nozzle with the outer-director compared to the original nozzle and the nozzle with the inner-director, indicating a larger area of medium turbulence intensity in the far field.

In addition, the centreline turbulence intensity for different nozzles are presented in Figure 6d. It can be seen that, in the near field, the centreline turbulence intensity for the nozzle with the inner-director generally resembles that for the original nozzle. While in the far field, due to the sharp decrease of turbulence intensity for the original nozzle, the turbulence intensity for the nozzle with the outer-director reaches a plateau similar to that for the original nozzle, indicating a similar level of velocity fluctuation for these two nozzles in the far field.

By presenting the measured turbulence intensity for the original nozzle with the simulated modeling result calculated by Krutka et al. [12], the trends of the experiment’s and CFD’s results can be compared. It can be seen from Figure 6d,e that, even though the experiment results and the CFD results are of a different scale, the turbulence intensity obtained through both methods share a similar trend. The peak of the centreline turbulence intensity shows up at some distance as the jet flow downstream, indicating the maximum perturbation within this area.

### 3.3. Fluctuation Intensity of Temperature

**Figure 7:**
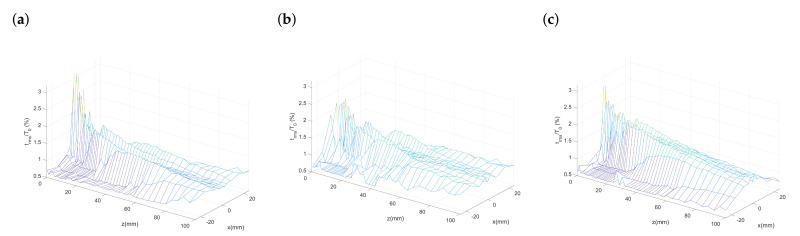
Mesh plots of temperature fluctuation intensity (TFI): (**a**) Original nozzle, (**b**) nozzle with the outer-director, and (**c**) nozzle with the inner-director.

In addition to the fluctuating velocity fields, the fluctuating temperature fields for different nozzle configurations are also obtained through our experiment as shown in Figure 7. Temperature fluctuation intensity is calculated by the ratio of the root-mean-square of temperature fluctuations $T_{rms}$ and the nozzle exit temperature $T_{0}$, where $T_{rms}=\sqrt{\frac{1}{N-1}\sum_{i=1}^{N}{\left(T_{i}-T_{mean}\right)}^{2}}$. In Figure 7a–c, the height of each mesh plot, which represents the peak value of the temperature fluctuating intensities, can be observed and compared. Unlike the nodes in mesh plots of turbulence intensity, the nodes representing temperature fluctuation intensity distributes in a much bumpier manner and a smaller peak value in the *x*–*z* plane. This means the fluctuating temperature field for the melt-blowing airflow are milder and yet irregularly distributed compared to the fluctuating velocity field. Moreover, the peak value for the nozzle with the outer-director is lower compared with the original nozzle and the nozzle with the inner-director. This represents the outer director can help a less intensified fluctuating temperature field near the nozzle exit.

**Figure 8:**
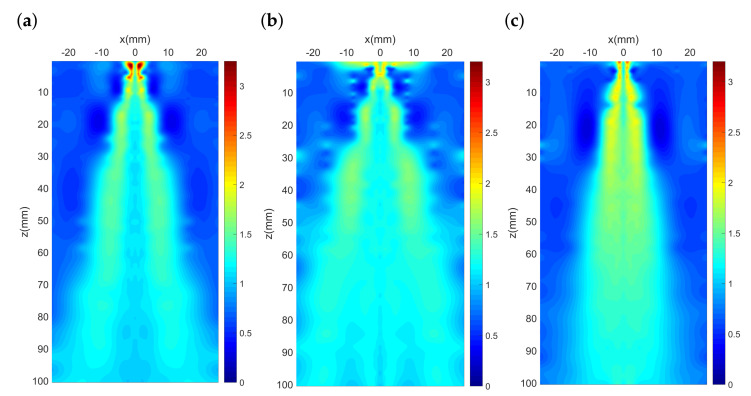
Distribution plots of temperature fluctuation intensity (TFI): (**a**) Original nozzle, (**b**) nozzle with outer-director, and (**c**) nozzle with inner-director.

Furthermore, the distribution plots of temperature fluctuation intensity (TFI) for the nozzles are obtained through biharmonic spline interpolation as shown in Figure 8. Unlike the peak location of turbulence intensity, the peak of temperature fluctuation intensity appears closer to the nozzle exit (0 mm<z<5 mm), indicating a sharper decrease for the fluctuating temperature as the air-jet blows downstream from the nozzle exit. In addition, Figure 8 shows that, for all the nozzles, the distribution plots share a pattern that the position of large value of temperature fluctuation intensity distributes at both sides away from the centreline rather than at the centreline. Based on the analyses in our previous work [28], we believe this distribution pattern can be attributed to the intense thermal instability driven by natural convection at this thermally unstable area. By comparing Figure 8b,c with Figure 8a, it can be found that the nozzle with an outer airflow-director possess a smaller temperature fluctuation intensity around the centreline. This manifests the stabilization of temperature at positions near the centreline during melt-blowing.

## 4. Effect of Airflow-Directors on Fibre Oscillations

It was assumed in the literature that the non-axisymmetric motions (whipping-like motions) of fibre are associated with turbulent air [6,35]. Thus, it is expected that fibre whipping (and its associated defects) could be reduced through modified die designs, which produce a modified airflow field to help stabilize too violent fibre motions. In our previous work, the effect of temperature on fibre bending instabilities has been investigated [28].

In this work, to investigate the influence of melt-blowing slot-die nozzle configurations on fibre motion characteristics, a 0.5 denier polyester filament fibre with a length of 10 cm was placed under the melt-blowing slot-die nozzles, where the fibre was subjected to blowing air at 200 m/s under constant temperature. The images of fibre motion were captured through a high-speed camera (i-SPEED 7, iX Cameras Ltd., Essex, U.K.) with a rate of 2000 frame/s. This method is capable of observing the fibre oscillation characteristics under the high-speed turbulent airflow field. Similar experiments of the fibre motion analysis can be found in the previous publication of Yarin et al. [13] and Zeng et al. [28].

**Figure 9:**
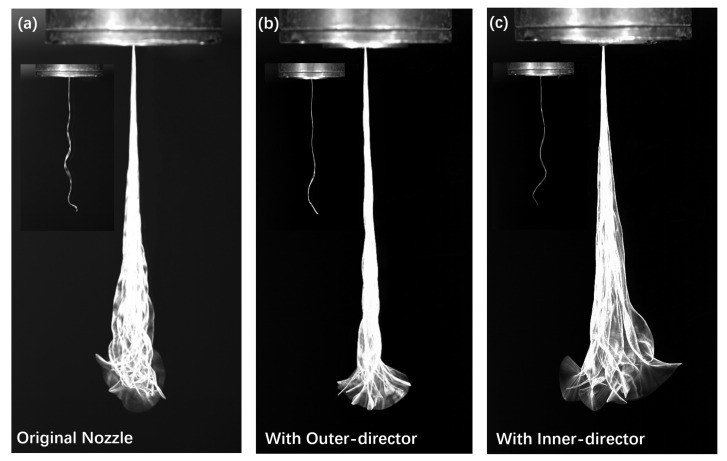
Overlapping of successive high-speed images of a filament fibre oscillation under the melt blowing slot-die nozzle: (**a**) Original nozzle, (**b**) nozzle with outer-director, and (**c**) nozzle with inner-director.

In Figure 9, the successive high-speed images for different slot-die nozzles, where each represent the overlaying of 100 photographs during 0.5 s of photography, are presented. To be mentioned, an example of the instantaneous image for each nozzle is also shown in the top-left of Figure 9a–c. Although the fibre seems straight near the nozzle exit, it can be seen clearly in the instantaneous images that the fibre oscillation arise as soon as the fibre leaves the nozzle instead of at some distance further downstream. It was pointed out in the literature that smaller wavelengths can be associated with larger whipping frequencies, which can lead to a more violent motion of fibre under the melt-blowing die [34]. To be mentioned, the oscillation wavelength is defined as the distance between successive crests in the instantaneous image of fibre. We can find in Figure 9 that the fibre for the original nozzle and the nozzle with inner-director contain a larger portion of smaller wavelength oscillations compared to that of the nozzle with the outer-director. Besides, the fibre oscillation for the nozzle with the outer-director is milder compared with that for the other two nozzles, indicating the suppression of fibre oscillation with the add-on of the outer-director under the melt-blowing slot-die nozzle.

**Figure 10:**
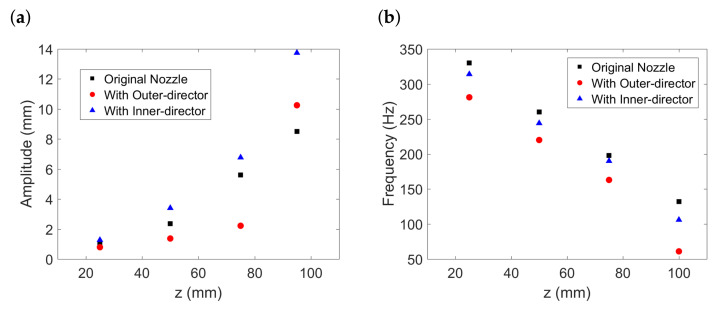
(**a**) Fibre oscillation amplitude along *z* and (**b**) fibre oscillation frequency along *z*.

In addition, considering the large bending instability of fibre that arises as soon as it exposed to high-speed turbulent airflow field, we expect the control of the near field turbulence has a great impact on the fibre bending instabilities, including not only fibre bending amplitude but also fibre bending frequency. Thus, we further calculated the amplitude and frequency of fibre oscillation along different *z*-positions according to the successive images (Figure 9), with the results presented in Figure 10. The amplitude of fibre bending instability is determined by the envelopes of the fibre movement for different nozzles at different positions, while the frequency of fibre bending instability is determined by calculating the movement of oscillating fibre at each *z*-position.

It can be seen in Figure 10a that the tendency within 80 mm is clear that the nozzle with the outer-director can restrict the fibre whipping amplitude compared to the other two nozzles. This indicates a strong drag force due to the air concentration near the centreline and can restrict the lateral excursion of fibre. On the other hand, as for the dramatic amplitude increase beyond 80 mm for the nozzle with the outer-director, we believe this can be explained as vigorous ‘flapping’ [33]. This phenomenon can be attributed to the turbulence energy in the almost unloaded part being released as kinetic energy and form lateral excursions. This can be helpful to a more evenly distribution of fibre during melt-blowing. It can be seen in Figure 10b that, fibre bending frequency for the nozzle with the outer-director is of a smaller value compared to the other two nozzle configurations. We believe this can be attributed to the control of turbulence intensity near the nozzle exit. Both the result of the fibre oscillation amplitude and frequency suggest the nozzle with the outer-director can create a more stabilized flow and and a milder fibre oscillation pattern.

## 5. Conclusions

In the present work, two new nozzle configurations with air-flow directors were designed. The effects of air-flow directors on time-averaged characteristics, turbulence intensity, and temperature fluctuation intensity were carried out by comparing their result with that of the original melt-blowing slot-die nozzle.

The result of the time-averaged characteristics showed that the existence of an outer airflow-director could help maintain both the centreline velocity and centreline temperature at a higher value at some distance downstream compared with the original nozzle. Thus, it could form an airflow field with stronger drag force and an superior temperature condition for the melt-blowing. The result of the turbulence intensity showed that the turbulence intensity near the nozzle exit (2.5 mm<z<12 mm) for the nozzle with the outer-director was of the lowest value among the conditions measured, while the turbulence intensity for the nozzle with the inner-director was considerably larger than that of the nozzle with the inner-director. In addition, the nozzle with the outer-director also produced a milder temperature fluctuation intensity. It is suggested that both turbulence intensity and temperature fluctuation intensity could be reduced by applying an airflow field with minimized spreading character. Finally, the effect of airflow-directors on the fibre oscillation wavelength, fibre bending amplitude, and fibre bending frequency was also discussed. It was further verified that the violent fibre motion could be tempered by applying blowing air with minimized spreading character. Thus, the results suggest that the existence of airflow directors, in spite of the lengths of the airflow directors being minimized, could strongly influence the distribution of turbulence. as we intend to avoid the contact between the oscillating fibre and the solid boundaries. This work provides an insight that the change of nozzle configuration can help control the pattern of fibre oscillation and may help improve melt-blowing fibre evenness.

## Data Availability

The data presented in this study are available on request from the corresponding author.
